# Nitric Oxide Synthase Blockade Impairs Spontaneous Calcium Activity in Mouse Primary Hippocampal Culture Cells

**DOI:** 10.3390/ijms24032608

**Published:** 2023-01-30

**Authors:** Rostislav A. Sokolov, David Jappy, Oleg V. Podgorny, Irina V. Mukhina

**Affiliations:** 1 Institute of Biology and Biomedicine, Lobachevsky State University of Nizhny Novgorod, 603022 Nizhny Novgorod, Russia; 2 Institute of Translational Medicine, Pirogov Russian National Research Medical University, 117513 Moscow, Russia; 3 Shemyakin-Ovchinnikov Institute of Bioorganic Chemistry, 117997 Moscow, Russia; 4 Institute of Fundamental Neurology, Federal Center of Brain Research and Neurotechnologies, Federal Medical Biological Agency, 117997 Moscow, Russia; 5 Center for Precision Genome Editing and Genetic Technologies for Biomedicine, Pirogov Russian National Research Medical University, 117513 Moscow, Russia; 6 Central Research Laboratory, Privolzhsky Research Medical University, 603005 Nizhny Novgorod, Russia

**Keywords:** nitric oxide, hippocampus, primary culture, calcium signaling, imaging

## Abstract

Oscillation of intracellular calcium concentration is a stable phenomenon that affects cellular function throughout the lifetime of both electrically excitable and non-excitable cells. Nitric oxide, a gaseous secondary messenger and the product of nitric oxide synthase (NOS), affects intracellular calcium dynamics. Using mouse hippocampal primary cultures, we recorded the effect of NOS blockade on neuronal spontaneous calcium activity. There was a correlation between the amplitude of spontaneous calcium events and the number of action potentials (APs) (Spearman R = 0.94). There was a linear rise of DAF-FM fluorescent emission showing an increase in NO concentration with time in neurons (11.9 ± 1.0%). There is correlation between the integral of the signal from DAF-FM and the integral of the spontaneous calcium event signal from Oregon Green 488 (Spearman R = 0.58). Blockade of NOS affected the parameters of the spontaneous calcium events studied (amplitude, frequency, integral, rise slope and decay slope). NOS blockade by Nw-Nitro-L-arginine suppressed the amplitude and frequency of spontaneous calcium events. The NOS blocker 3-Bromo-7-Nitroindazole reduced the frequency but not the amplitude of spontaneous calcium activity. Blockade of the well-known regulator of NOS, calcineurin with cyclosporine A reduced the integral of calcium activity in neurons. The differences and similarities in the effects on the parameters of spontaneous calcium effects caused by different blockades of NO production help to improve understanding of how NO synthesis affects calcium dynamics in neurons.

## 1. Introduction

Nitric oxide (NO) is a small molecule that is a secondary gaseous messenger [1]. NO is crucial for various biological systems, including the central and peripheral nervous, cardiovascular, immune and reproductive systems [2,3,4,5]. Under physiological conditions, it contributes to regulating proliferation, survival and neuronal differentiation [6]. Three nitric oxide synthase (NOS) genes whose products have distinct tissue localization and properties are known: neuronal, inducible and endothelial NOS (nNOS, iNOS, and eNOS, respectively) [7]. nNOS participates in the regulation of neurogenesis processes presumably preventing cell proliferation, participating in memory formation, learning processes, sexual behavior, excitotoxicity and a number of neurodegenerative conditions [7,8]. NO released from a small subpopulation of NOS-containing neurons that lie in apposition to cerebral arterioles appears to be involved in mediating the coupling between neuronal activity and cerebral blood flow and metabolism [9].

nNOS is located in the cytoplasm of a small subpopulation of γ-aminobutyric acid (GABA)-ergic neurons in the cortex and hippocampus and is also located discretely at the post-synaptic density (PSD) in pyramidal cell spines [10]. Synaptic localization is regulated by the n-terminus of nNOS, which encodes a PDZ protein motif that interacts with PSD-95/PSD-93 through a PDZ–PDZ binding interface [11].

Activation of eNOS and nNOS is Ca^2+^-calmodulin-dependent (CaM), and thus depends on [Ca^2+^]_in_ [12]. Several putative phosphorylation sites have been identified, allowing for modulation of nNOS by cyclic adenosine monophosphate (cAMP)- and cyclic guanosine monophosphate (cGMP)-dependent kinases (Protein Kinase A (PKA) and Protein Kinase G (PKG), respectively). Protein Kinase A (PKC) and Ca^2+^/calmodulin-dependent protein kinase II (CaMKII) also modulates nNOS [13,14]. In the rat hippocampus, serine-847 phosphorylation is affected by glutamate stimulation in a biphasic manner: low concentrations (low mM range) increased and high concentrations (100 mM) decreased activity via a CaMKII-dependent pathway, causing a 50% reduction in NO generation [15,16,17]. CaMKII phosphorylates nNOS, reducing its activity, but serine/threonine protein phosphatase 1 dephosphorylation increases NO production [7]. Another serine/threonine protein phosphatase 2B (PP2B, calcineurin) can directly dephosphorylate PKC-dependent or CaMKII-dependent phosphorylation of nNOS at serine-847, enhancing its activity [18,19]. α-amino-3-hydroxy-5-methyl-4-isoxazolepropionic acid receptor (AMPAR) trafficking was shown to be dependent on calcineurin [20]. Another way in which [Ca^2+^]_in_ regulates nNOS is via the interaction of the plasma membrane calcium/calmodulin-dependent calcium ATPase 4b (PMCA4b) with the nNOS PDZ domain [21]. An increase in [Ca^2+^]_in_ causes increased protein–protein interactions between PMCA4b and nNOS, forming a complex; when PMCA is active, the Ca^2+^ concentration decreases, also decreasing the activity of nNOS, thus PMCA4b may be involved in nNOS deactivation [22].

NO can contribute to both anterograde and retrograde signaling at the synapse, making it uniquely suited to mediate induction of hippocampal long-term potentiation (LTP) [23,24,25]. NO may influence synaptic efficacy by regulating neurotransmitter release [2,11], and the formation of short-term memory is a NO/NOS-dependent process [26]. Stimulation of soluble guanylyl cyclase (sGC) by NO leads to cGMP generation and activates the PKG-dependent signaling necessary for various signaling cascades and short-term synaptic plasticity [27]. Due to the indirect spread of NO, the targets of nitrergic signaling pathways are diverse and often unresolved.

Changes in [Ca^2+^]_in_ are inherent in all types of cells [28,29,30]. These changes exert an influence on all cellular physiological functions (activation of calcium-dependent kinases, phosphatases, gene expression, bioenergetics, etc.) [31,32]. Calcium-permeable receptors and channels on the cell membrane, intracellular calcium stores, protein signaling molecules and NOS are involved in the formation of intracellular calcium waves [33]. NO, as a product of NOS, can affect calcium dynamics and synaptic efficacy by regulating neurotransmitter release in neurons [10,34]. However, the activity of voltage-gated calcium, voltage-gated potassium, Ca^2+^-activated, ATP-sensitive potassium, hyperpolarization-activated cyclic nucleotide-gated (HCN), leak channels and interactions with other signaling pathways (such as metabotropic glutamate receptors, endocannabinoids, and catecholamine receptors) might be modulated via NO nitrosylation [35,36,37,38,39,40,41,42].

Here, we evaluated the effect of NOS blockade on spontaneous calcium activity in cultured neurons from the hippocampus of mouse embryos using different NOS blockers and a blocker of NOS upregulation. We assessed the amplitude, frequency and integral of spontaneous calcium events (SCEs) and determined the contribution of the NOS-upregulating protein calcineurin to spontaneous calcium activity in neurons. Since the different targets of blockade in this study have different localizations and properties, similarities or differences in their effects will help improve understanding of calcium dynamics in neurons.

## 2. Results

### 2.1. NO Synthesis in Hippocampal Primary Cultures

Dissociated primary neuronal cultures are widely used as a model system to investigate the cellular and molecular properties of diverse neuronal populations and the mechanisms of action potential generation and synaptic transmission. Hippocampal cultures show all the features of neuronal activity, such as formation of synchronous burst activity and spontaneous calcium oscillations, and they share many key properties with CA1 pyramidal cells during long term potentiation (LTP) [43,44].

**Figure 1:**
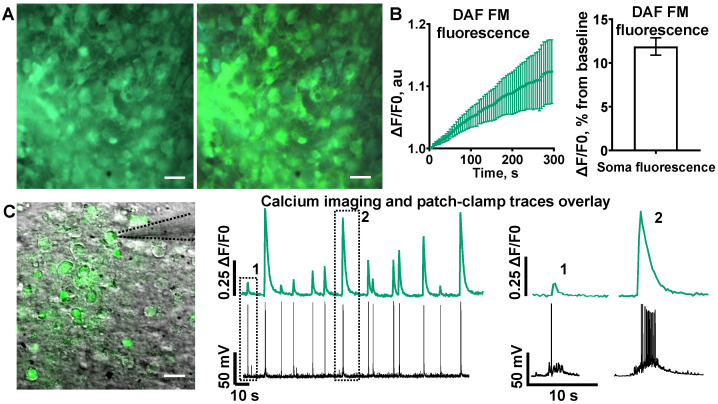
The relationship between number of action potentials, amplitude of calcium events and nitric oxide synthesis. (**A**) Two photos of DAF-FM loaded cells with a 5 min gap. Scale bar—20 μm. (**B**) Left: fluorescent increase in DAF-FM over 5 min of acquisition. Right: average increase in DAF-FM fluorescence in cell somas after 5 min. (**C**) Left: Oregon Green-brightfield overlay. Black dots show the patch pipette. Middle and right: superposition of calcium imaging trace and patch-clamp trace in current clamp mode from the same cell, with time magnification of single calcium events of different amplitudes.

We recorded the increase in the amount of nitric oxide in neuronal cell bodies (Figure 1A). DAF-FM fluorescent emission simply increased linearly, showing the rise of NO concentration with time in soma (Figure 1B, *n* = 30, 11.9 ± 1.0%, 5th min). NO is a vital second messenger in cultured neuronal networks and is necessary for network activity.

### 2.2. Spontaneous Electric Activity in Cultured Neurons Is Closely Related to Calcium Oscillations

**Figure 2:**
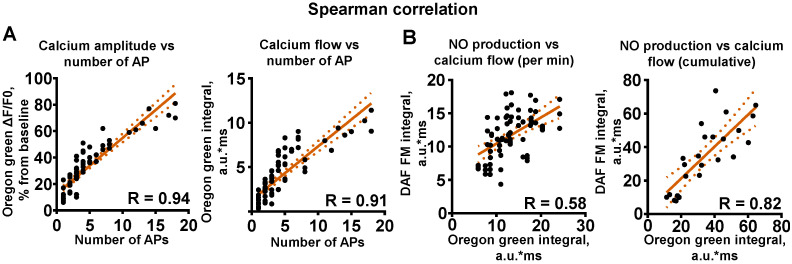
Spearman correlation analysis of action potentials, calcium events and nitric oxide production. (**A**) Left: correlation of SCE amplitude and number of action potentials in bursts. Right—correlation of SCE integral (arbitrary units * milliseconds) and number of action potentials in bursts. (**B**) Left: correlation of sum of integrals calculated for DAF-FM and Oregon Green for 1 min. Right: correlation of cumulative sum for integrals for 1–5 min of recording.

Spontaneous activity in cultures varies from single APs to AP bursts of different sizes [45]. Concurrent recording of neuronal firing via patch-clamp and calcium fluorescent imaging showed action potential (AP)-associated calcium activity (Figure 1C). The amplitude of SCEs was related to the number of APs in the burst. Spearman correlation analysis showed high codependency (Figure 2A, *n* = 5, R = 0.94). The integral of SCEs can be interpreted as the total change in [Ca^2+^]_in_ in the cytosol over time, or calcium flow throughout the cytosol. In addition to SCE amplitude, we evaluated the correlation of the amount of APs and the SCE integral (Figure 2A, *n* = 5, R = 0.91). Thus, it can be concluded that the indicators of the amplitude and OG integral of SCEs depend on the number of APs generated by the cells.

To assess the relationship between NO synthesis and calcium activity in neurons, we performed a correlation analysis of NO production and [Ca^2+^]_in_ by measuring DAF-FM and OG fluorescence. We used recordings from different neuronal cultures, since the fluorescence emission spectra of OG and DAF-FM overlap. Fluorescence measurements were performed for 5 min. Minute-by-minute evaluation using Spearman correlation analysis showed a rather high degree of correlation between NO increase and calcium integrals (Figure 2B, *n* = 6, R = 0.58). The cumulative calculations of OG integral and NO production from the 1st to the 5th minute also indicated a high correlation over a longer period (Figure 2B, *n* = 6, R = 0.82). These correlations clearly indicate a link between intracellular calcium and NO, and thus involvement of NO in the regulation of spontaneous neuronal activity.

### 2.3. Effect of NOS-Blockade on Calcium Activity Is Associated with Spontaneous Neuronal Firing

**Figure 3:**
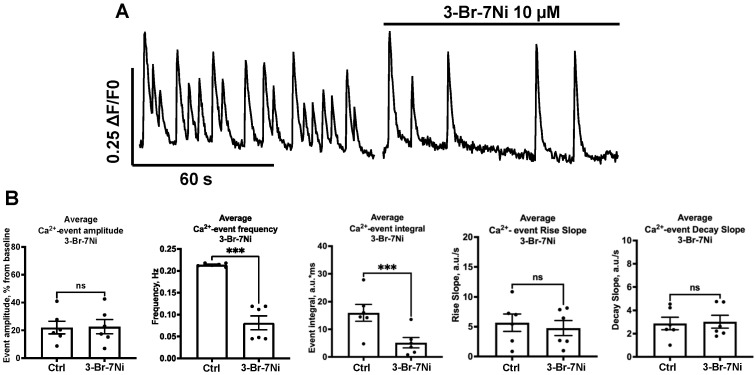
Influence of the 3-Br-7Ni blocker on SCEs. (**A**) Typical recording of the influence of 3-Br-7Ni on SCEs. (**B**) Normalized amplitude (not significant), frequency (*p* *** = 0.001), integral (*p* *** = 0.0009), rise slope (not significant) and decay slope (not significant) for these influences are shown.

NO participates in many intracellular processes linked to calcium signaling and neurotransmission. Considering there is NO synthesis in hippocampal primary cell cultures, we tested how NO synthesis blockade contributes to the calcium activity associated with spontaneous neuronal firing. Two different NOS blockers were used in the study. Nw-Nitro-L-arginine (L-NNA 100 μM; Sigma Aldrich, St. Louis, MI, USA) is a blocker of all NOS isoforms, and 3-Bromo-7-Nitroindazole (3-Br-7Ni 10 μM; Tocris, Bristol, UK) is a selective blocker of the nNOS isoform. For 3-Br-7Ni, the average amplitude did not change (Figure 3B, *n* = 6, 22.00 ± 4.55 vs. 22.67 ± 5.16%, n.s.). The average frequency decreased significantly (Figure 3B, *n* = 6, 0.21 ± 0.01 vs. 0.08 ± 0.02 Hz, *p* = 0.001). The calcium integral also significantly decreased (Figure 3B, *n* = 6, 15.9 ± 3.0 vs. 5.2 ± 1.9 a.u.*ms, *p* = 0.0009). Two-fold reduction in the [Ca^2+^]_in_ integral indicates powerful suppression of calcium-conducting systems. At the same time, the rise slope (20–80%) showed no significant increase (Figure 3B, *n* = 6, 5.7 ± 1.5 vs. 4.8 ± 1.3 a.u./s, n.s.), as well as the decay slope (0–50%) (Figure 3B, 2.9 ± 0.5 vs. 3.0 ± 0.6 a.u./s, n.s).

**Figure 4:**
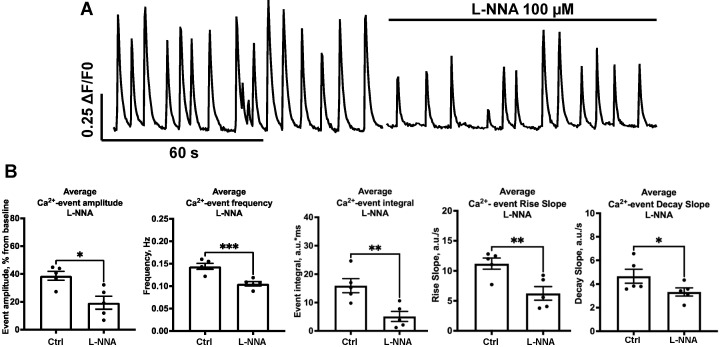
Influence of the L-NNA blocker on SCEs. (**A**) Typical recording of the influence of L-NNA on SCEs. (**B**) Normalized amplitude (*p* * = 0.026), frequency (*p* *** = 0.0001), integral (*p* ** = 0.0091), rise slope (*p* ** = 0.0088) and decay slope (*p* * = 0.033) for these influences are shown.

**Figure 5:**
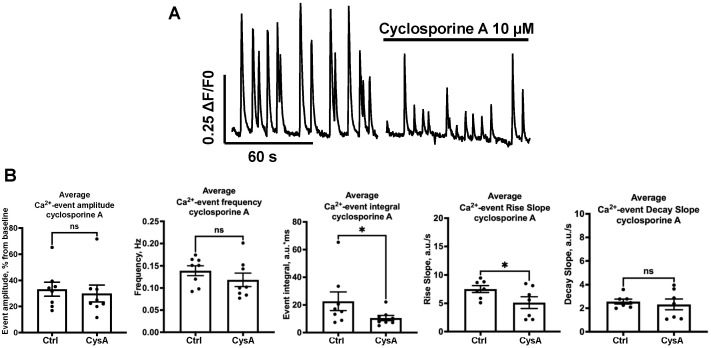
Influence of cyclosporine A on SCEs. (**A**) Typical recording of the influence of cyclosporine A on SCEs. (**B**) Normalized amplitude (not significant), frequency (not significant), integral (*p* * = 0.045), rise slope (*p* * = 0.046) and decay slope (not significant) for this influence are shown.

Unlike 3-Br-7Ni, L-NNA significantly decreased the average amplitude of events (Figure 4B, *n* = 5, 38.70 ± 3.20 vs. 19.33 ± 4.65%, *p* = 0.026). The average frequency of events also decreased (Figure 5B, *n* = 5, 0.14 ± 0.01 vs. 0.11 ± 0.01 Hz, *p* = 0.0001). The calcium integral significantly decreased and was very close to that of 3-Br-7Ni (Figure 4B, *n* = 5, 15.9 ± 2.5 vs. 5.1 ± 1.7 a.u.*ms, *p*=0.0091). The rise slope (Figure 4B, *n* = 5, 11.20 ± 0.9 vs. 6.2 ± 1.1 a.u./s, *p* = 0.0088) and decay slope (Figure 4B, *n* = 5, 4.7 ± 0.6 vs. 3.3 ± 0.4 a.u./s, *p* = 0.033) of the calcium events significantly decreased with L-NNA, indicating changes in [Ca^2+^]_in_ dynamics.

Calcineurin-dependent upregulation of NOS activity and NO production is well-known. We therefore examined how the calcineurin blockade affects SCEs for further estimation of the activity of NO production. Cyclosporine A (CysA 10 μM; Tocris, Bristol, UK), a well-known blocker of calcineurin, did not affect the SCE amplitude (Figure 5B, *n* = 8, 33.22 ± 5.37 vs. 29.92 ± 6.48%, n.s.). However, the frequency of SCEs showed no significance (Figure 5B, *n* = 8, 0.139 ± 0.01 vs. 0.118 ± 0.02 Hz, n.s.). The integral decreased twofold (Figure 5B, *n* = 8, 22.7 ± 6.7 vs. 10.5 ± 1.9 a.u.*ms, *p* = 0.045). The parameters of the rise slope (Figure 5B, *n* = 8, 7.5 ± 0.6 vs. 5.1 ± 1.0 a.u./s, *p* = 0.046) and decay slope (Figure 5B, *n* = 8, 2.6 ± 0.2 vs. 2.3 ± 0.5 a.u./s, n.s.) of SCEs significantly decreased.

The results indirectly indicate a strong relationship between phosphatase–kinase balance and the calcium activity of neurons. Thus, blocking NOS activity in primary hippocampal cell cultures causes suppression of calcium activity.

## 3. Discussion

This study is devoted to the dependence of spontaneous AP-associated calcium activity on NO/NOS activity in hippocampal neuron-glial cultures. We evaluated AP-associated calcium activity and found that different blockades of NOS have similar and different effects on calcium dynamics, allowing us to make hypotheses as to why.

Concurrent patch-clamp and imaging revealed a direct correlation between the number of APs in bursts and fluorescence intensity. The correlation between the number of APs and calcium increase was established for the amplitude and integral parameters of SCEs. Changes in SCE amplitude may indirectly indicate changes in the activity of AP-related molecular targets. This link is mostly related to synaptic targets and voltage-dependent channels [46].

The linear increase in DAF-FM fluorescence indicates stable somatic NO production. We evaluated the correlation of OG and DAF-FM integrals. The coefficient obtained from the analysis is evidence for this connection. In soma, voltage-dependent calcium channels (VDCCs) and ryanodine receptors (RyRs) play an important role in increasing intracellular calcium concentration in response to external and internal stimuli. NO participates in the regulation of these systems, and the NOS blockade led to a reduction in AP-associated calcium activity. Due to the dual role of NO and its participation in physiological and pathological effects, the issue of increased neuronal activity due to increased NO production needs to be resolved in the future. Based on the results, an increase in the rates of spontaneous calcium events and action potentials are expected.

nNOS is mostly located in the post-synapse, which can explain the difference in the effect of 3-Br-7Ni and L-NNA on the amplitude and frequency of SCEs from cellular somas. The nNOS blockade affects the frequency of SCEs to a greater extent than amplitude, since nNOS is mostly localized in synapses [10,11]. Neuronal network activity depended on nNOS blockade, while not affecting the amplitude of SCEs on neuronal somas. A decrease in the frequency of SCEs when blocking NOS is associated with the need for the presence of NO to maintain calcium homeostasis in synapses [47]. For example, PMCA4b is one of the major players in calcium balancing; it interacts with nNOS at the PDZ site reducing NO production [21]. The dose-dependent manner of L-NNA action may also be an answer to the question about the difference between the effects of L-NNA and 3-Br-7Ni on the frequency of SCEs [48].

NOS blockade decreased the frequency, amplitude and integral of SCEs in neurons. This indirectly indicates reduced neuronal excitability. At the postsynapse, ionotropic and metabotropic glutamate receptors (NMDARs, AMPARs, mGluRs) are affected by NO [49]. As a consequence, postsynaptic activation may suffer in the absence of NO. An additional explanation of the SCE frequency decrease might be connected to NO-dependent trafficking and expression of AMPARs on the postsynapse [20]. The absence of NO affects the activity of mGluRs and G-proteins, reducing the excitability of the postsynaptic membrane [50]. Insufficient calcium activity of neuronal soma in the presence of L-NNA due to reduced postsynapse excitability may be the cause of insufficient presynaptic vesicular release. Normally, APs from the soma arrive at the presynapse leading to the activation of VDCCs, RyRs and, as a consequence, calcium-dependent presynaptic processes: vesicular transport and neurotransmitter release [51]. NO also enhances calcium release from intracellular stores [52,53]. S-nitrosylation plays a role in the activation of L-type VDCCs, and increases the release of glutamate. Blocking NO synthesis affected the presynaptic targets and the processes of presynaptic function associated with them. This may explain the decrease in the frequency of SCEs, which reflect network activity, when blocking the synthesis of NO. Interestingly, both 3-Br-7Ni and L-NNA reduced the integral to similar values that might be a threshold calcium flow level, which neurons can maintain without NO activity. The rise and decay slopes mostly reflect the speed of calcium propagation with calcium intake channels and calcium removal systems (PMCA and SERCA). The absence of rise and decay slope changes with the nNOS blocker 3-Br-7Ni reveals no changes in calcium intake and calcium removal. This might be connected to the strong balancing mechanisms of the calcium amount for intracellular calcium-dependent processes. With L-NNA, we see the influence on the rise and decay slope. It changes, meaning a longer front of calcium intake and slower calcium removal from the soma. This may be due to overfilled calcium stores. RyRs cannot act normally without nitrosylation, leading to a reduction in calcium leaving the endoplasmic reticulum (ER) via RyRs, lowering SERCA activity because the calcium concentration in the ER remains high [51].

There is no NO-dependent decrease in SCE frequency when blocking calcineurin synaptic activity [24]. CysA did not affect SCE amplitude and frequency, showing its activity is mostly related to postsynaptic rather than somatic calcineurin targets. Calcineurin also showed lower calcium flow control than NOS, but the rise slope changed in the same direction as in the presence of L-NNA. This might indicate some downregulation of calcium intake but not removal when calcineurin is blocked [54]; however, calcineurin has a number of other effects on synaptic physiology.

These results reveal the calcium dynamics in relation to NO production in neurons, and lead to hypotheses that must be further investigated.

## 4. Materials and Methods

### 4.1. Cell Culture Preparation

**Figure 6:**
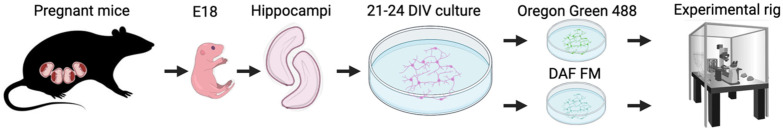
Time-line diagram of experimental procedures. This time-line diagram shows the pregnant mice at E18 day of embryos’ development. Embryos were dissected from uterus. The hippocampi were dissected from embryos and cut. Plated cultures grew until the 21–24 DIV, and then were incubated for 40 min with Oregon Green 488 or DAF-FM dyes. After incubation, cultures were placed in the recording chamber and moved to experimental rig.

A mixed C57Bl/6 mouse primary embryonic neuronal cell culture was prepared as described in [46], with the protocol number 357 for animal handling and cell culture preparation approved by the Ethics Committee of the Shemyakin-Ovchinnikov Institute of Bioorganic Chemistry. Mice husbandry included breeding pairs in the cage ad libitum. For neuron-glial culture preparation and cultivation, Thermo Scientific mediums and supplements (Gibco, New York, NY, USA) were used. Briefly, pregnant mice at stage E18 were euthanized with commonly used isoflurane and then decapitated; the uterus was dissected out and the embryos were placed in a sterile Petri dish with Hanks’ solution. Dissected hippocampi were collected in a tube with pre-heated Versen solution. Cut hippocampi were trypsinized with 0.31% solution for 15 min in an incubator. After incubation, trypsin was removed and cells on the bottom were rinsed with Plating medium containing DMEM, 10% FBS and 1% gentamicin (Sigma-Aldrich, St. Louis, MO, USA). Next, 500 μL Plating medium with 40 μL DNAse was added for 10–15 s. Washed cells were resuspended and counted in the Goryaev chamber. Polyethylene-imine-coated covers were placed in plate wells and 4 × 104 cells were plated on each. After 1 h incubation, the wells were filled with Full NBM I, containing Neurobasal media, 0.05% β-mercaptoethanol (Sigma-Aldrich, St. Louis, MO, USA), 1% Glutamax, 2% B27 supplement and 1% gentamicin (Sigma-Aldrich, St. Louis, MO, USA). After 7 days, the medium was replaced with Full NBM II, containing Neurobasal media, 1% Glutamax, 2% B27 supplement and 1% gentamicin (Sigma-Aldrich, St. Louis, MO, USA). Cultures were fed every 2–3 days. All experiments were conducted during the daylight (Figure 6).

### 4.2. Intracellular Calcium Recordings

Oregon Green-488 BAPTA-1 AM (OG; Invitrogen, Carlsbad, CA, USA) dissolved in dimethylsulfoxide (1 mM stock) was added to each culture before the experiment to a final concentration of 1 μM. Cultures were incubated for 40 min at 37 °C, CO_2_ 5%. Then, coverslips were submerged in a recording chamber. The recording chamber was continuously perfused at 1.5 mL/min with a solution containing (mM): 130-NaCl, 2.5-KCl, 1.5-MgCl_2_, 1.5-CaCl_2_ 10-glucose, 10-HEPES, 300 ± 5 mOsm, pH-7.33. An Evolve512 EMCCD camera (Photometrics UK Ltd., Marlow, UK), mounted on the SliceScope Pro 2000 (Scientifica, Uckfield, UK) microscope, was used (Figure 6). Data acquisition was made with 3.6 fps. ROI positioning and dF/F0 analysis were performed with AxioVision Imaging System (Carl Zeiss, Jena, Germany). Calcium events were analyzed using Mini Analysis Program (Synaptosoft, Decatur, GA, USA), and pClamp (Molecular Devices, San Jose, CA, USA). The baseline of Ca^2+^-sensitive fluorescence for each cell at rest (between two neighboring calcium events) was taken as a 100% reference point. Thus, the amplitude of each SCE is represented as n% from the baseline. We evaluated the following SCE parameters: amplitude, frequency, individual integral, total integral (sum of integrals), rise slope and decay slope. The SCE calculations were carried out on a 5 min recording segment for control and 5 min recording segment for tested blockers, with the 5 min gap after the blockers were added to the recording solution. Arbitrary units for integrals and slopes were presented in tenths. Names of used chemicals and blockers with the catalogue number are showed in Table 1.

### 4.3. Intracellular NO Recordings

DAF FM is almost nonfluorescent until it reacts with NO; DAF FM is a dye that irreversibly binds to NO. The fluorescence quantum yield of DAF-FM is ∼0.005, but increases about 160-fold, to ∼0.81, after reacting with NO. DAF-FM Diacetate (Invitrogen, Carlsbad, CA, USA) was dissolved in dimethylsulfoxide (1 mM stock) and added to each culture before the experiment to a final concentration of 1 μM. Cultures were incubated for 40 min at 37 °C, CO_2_ 5%. Then, coverslips with cultures were submerged in a recording chamber. The recording chamber was continuously perfused as described above, and the microscopy setup was the same as the above (Figure 6). Data acquisition and ROI positioning were performed with AxioVision Imaging System (Carl Zeiss, Jena, Germany). The NO signal was analyzed using pClamp (Molecular Devices, San Jose, CA, USA). The calculation of DAF-FM fluorescence was carried out on a 5 min recording segment with 1 frame per 5 s. Arbitrary units for integrals and slopes were presented in tenths.

### 4.4. Whole-Cell Patch-Clamp Recordings

Patch-clamp recordings of neurons were conducted at 21–26 DIV. Neurons were viewed under DIC or epifluorescence illumination at 40× magnification. Whole-cell patch-clamp recordings were obtained at room temperature (23–25 °C) using thin-walled glass pipettes (3–5 MΩ), filled with (mM): 140-K-gluconate, 1-MgCl2, 3-L-ascorbic acid, 10-HEPES, 2.5-Na2ATP, 1-Na3GTP, 295 ± 3 mOsm, pH-7.35. Names of used chemicals with the catalogue number are showed in Table 1. Heka Elektronik EPC 10 USB Patch Clamp Amplifier was connected to a computer running Patchmaster (HEKA Elektronik GmbH, Ludwigshafen/Rhein, Germany). Data were collected with a 10 kHz digitization rate in current-clamp mode. Acceptable cells had resting potentials greater than −60 mV.

### 4.5. Statistical Analysis

All values are given as mean ± SEM from n-cells calculated for at least 4 separate cultures from 3 independent cell passages. For analysis, random spontaneously active cells from the field of view were taken. Data passed the Kolmogorov–Smirnov normality test; statistical analysis was performed using paired parametric *t*-test in Graphpad (Prism, San Diego, CA, USA). The difference between groups was considered significant at *p* < 0.05. No blinding was used.

## Figures and Tables

**Table 1:** Names of used chemicals with the catalogue number.

Name	Cat.#
Abcam GTP trisodium salt	ab146528
Applichem Calcium Chloride	A3652,1000
Applichem D-glucose	141341
Applichem Sodium Chloride	141659
Applichem Magnesium Chloride 6-hydrate	141396
Applichem Potassium Chloride	A2939,1000
Sigma Adenosine 5′-triphosphate disodium salt hydrate	A26209-5G
Sigma HEPES	H3375-250G
Sigma L-Ascorbic acid	A4544-25G
Sigma Potassium D-gluconate	G4500-100G
Tocris 3-Bromo-7-nitroindazole	0735
Sigma Nω-Nitro-L-arginine	N5501
Tocris Cyclosporine A	1101

## Data Availability

Data available upon reasonable request.
